# Orthodoxy and Dissent

**Published:** 1903-01-31

**Authors:** 


### The Hospital.

A Journal of

The Medical Sciences and Hospital Administration.

Vol. XXXIII.—No. 853. Edited by Sir Henry Burdett, K.C.B., and Solomon C. Smith, M.D., M.R.C.P. [January 31, 1903.

### Orthodoxy and Dissent.

It is well to see all sides of every question and by the exercise of a wide charity to find good in everything, even in those whose opinions we hold in small respect. It was then a somewhat happy thought which led Dr. Killick Millard in addressing the Society of Medical Officers of Health to seek out the good points in the character of the "anti"—that dissenter from all that is orthodox. It must be admitted that cranks, faddists, and medical dissenters are not generally beloved by those who practise medicine, chiefly perhaps because their methods of controversy run on a plane of their own which is not generally acceptable, and it is interesting to find that Dr. Millard, who is health officer for the borough of Leicester, and must therefore have had a good deal of personal experience of the creature he describes, is able to find so many good things to say about that curious person of whom he speaks in general terms as the "anti."

He says, as we think very truly, that the adherents of the various "anti" movements met with at the present day have certain special attributes in common, so much so that there may be said to exist a specific "anti" type of human character. There can, indeed, be no doubt that the man who dissents from one thing is quite likely to dissent from half a dozen. He who is in politics a radical—dissenting root and branch from the state of things he finds around him —is quite likely to be a nonconformist in religion, a homœopathist in regard to medicine, an anti-vaccinist, and an "anti" many other things which by law or custom are established. His knowledge of politics, theology, or science may be of the smallest, but his habit of mind leads him to be always "agin the Government," and indeed "agin" the majority of his fellows. Dr. Millard says. that in conversing with a thorough-going "anti" of his acquaintance, he had admitted that majorities were sometimes wrong. "Ah!" was the reply, "when you have lived as long as I have, you will have come to the conclusion that majorities are *always* wrong!" Here we have the man in a nutshell. Other characteristics are said to be "independence, enthusiasm, conviction, perseverance, self-reliance, self-denial," all of which speak well for him.

On the other hand, even Dr. Millard admits that the "anti" has his own special faults, egoism and self-conceit being amongst the most frequent, while by his enemies even his virtues are distorted and misnamed. What Dr. Millard calls his perseverance and determination they call obstinacy and bigotry; "his enthusiasm and self-denial they call 'fanaticism,' and his militancy they name 'aggression'; whilst as for his altruism, they explain it by ascribing to him a love of notoriety. They sum up his character by describing him as a 'crank,'" and, while admitting to the full that opposition is always useful, that it is only by constant and sometimes painful probings that we can discover truth, and that in this way the "anti" may have his uses, we cannot but feel that the description of him given by his enemy is at least as appropriate as that furnished by his friend. When we come to consider what Dr. Millard tells us as to the useful functions subserved by these strenuous gentlemen we cannot but feel that he attributes a little too much importance to certain "anti" movements which do but express in an exaggerated and sometimes grotesque form various tendencies of the times which the propagandists certainly did not initiate, and over the progress of which they have exercised but little influence. By instances culled from the various "anti" movements of the day, from vegetarianism, anti-vivisection, teetotalism, anti-vaccination, and Malthusianism, Dr. Millard endeavours to show what good these protestors against accepted views and these dissenters from the orthodox have done in their time and generation, and to prove that even when the "anti" is in error he may be accomplishing a very useful end by forcing us to investigate afresh the groundmarks of our beliefs. The latter virtue we cheerfully admit, although we could wish that he would be content to do his work with rather greater regard for the canons of controversy. It is all very well to decry intolerance as Dr. Millard does, and to warn us not to be too positive as to our being right and the "anti" wrong; but are we really so intolerant as all this suggests? Dr. Millard says: "Man has always been an intolerant animal, both before and after the Spanish Inquisition; and though the roasting of heretics is not now in fashion, the spirit which prompted it to some extent remains." Which may be true. But when he seeks to apply this to medicine, we ask are we not really flabby rather than intolerant? The fact is that the widest tolerance is nowadays displayed towards the most heterodox opinions on any and every subject. We not only tolerate the "anti" and his "crank" as natural products of the era in which we live, but we even accept them with more or less appreciation as a means of undermining dogma and breaking down crystallized opinion. What is not to be tolerated is the introduction of methods of controversy which tend to the obscuring of truth. But that is a side of the "anti" character which Dr. Millard has not touched upon; yet it is really at the root of much of the friction between orthodoxy and dissent, and of much of the irritation felt by medical men with regard to the "anti" and his ways.

